# The Strategic Management of Infected Pancreatic Pseudocyst in the Postpartum Period After Cesarean Delivery in a Pregnancy Complicated by Preeclampsia/Hemolysis, Elevated Liver Enzymes, and Low Platelets (HELLP) Syndrome and Shock

**DOI:** 10.7759/cureus.72009

**Published:** 2024-10-21

**Authors:** Irva M Cheema, Ahsan Shafiq, Roshan Butt, Fahad Yasin, Muddassir Shafiq

**Affiliations:** 1 Department of Surgery, Shalamar Institute of Health Sciences, Lahore, PAK; 2 Surgical Oncology, Shaukat Khanum Memorial Cancer Hospital and Research Centre, Lahore, PAK

**Keywords:** infected necrotizing pancreatitis, medical disorders in pregnancy, necrotising pancreatitis, pancreatic pseudocyst (ppc), pancreatits in pregnancy, post partum pancreatitis

## Abstract

Pancreatitis is a rare but serious condition in obstetric patients, associated with significant morbidity and mortality. It can range from mild acute pancreatitis to severe complications such as necrosis, abscesses, pseudocysts, and multi-organ failure. While hypertriglyceridemia is a common cause, pancreatitis has also been linked to preeclampsia and shock. We present the case of a 22-year-old primigravida who developed an infected pancreatic pseudocyst 27 days after a cesarean section performed at 35 + 1 weeks of gestation due to preeclampsia/hemolysis, elevated liver enzymes, and low platelets (HELLP) syndrome, complicated by disseminated intravascular coagulation (DIC) and shock. Initial CT scans revealed a large pseudocyst, which was managed conservatively with antibiotics and ultrasound-guided drainage. On the 46th postoperative day, the patient underwent a retroperitoneal incision and drainage. She was discharged three days after the procedure, and a follow-up two weeks later showed complete recovery and healing of the drainage wound. The current literature lacks established treatment guidelines for managing pancreatitis and its complications in obstetric patients. Based on this case and a review of previous reports, we recommend an approach that starts with conservative management, followed by minimally invasive procedures if necessary, for treating infected pancreatic pseudocysts in the postpartum period. Given that patients are already recovering from the stresses of pregnancy and delivery, avoiding highly invasive techniques that require additional anesthesia or surgery can help minimize stress and improve overall well-being.

## Introduction

Acute pancreatitis affects 5-80 cases per 100,000 pregnancies and, if not managed properly, can lead to severe complications such as organ failure and maternal or fetal death [1]. Unlike in the general population, where alcohol intake and gallstones are the most common causes of pancreatitis, acute pancreatitis in pregnancy is most commonly due to biliary disease and hypertriglyceridemia [2]. In rare instances, it can be associated with pregnancy-related conditions such as preeclampsia or hemolysis, elevated liver enzymes, and low platelets (HELLP) syndrome, which pose additional risks. Preeclampsia is characterized by high blood pressure and organ dysfunction, typically occurring after 20 weeks of pregnancy. HELLP syndrome, a severe form of preeclampsia, involves liver damage, low platelet counts, and red blood cell destruction. Both conditions can impair blood flow and contribute to complications such as pancreatitis [3-6]. 

Pancreatitis during pregnancy is especially significant due to its negative effect on gestation, including increased risk of preterm labor, prematurity, and maternal and fetal death [1]. Similarly, a delay in diagnosis of this condition during pregnancy leads to the development of complications like pancreatic pseudocyst (fluid collections that develop in or around the pancreas after inflammation), thrombosis, hemorrhage, or infection, which further complicate management. Furthermore, during the postpartum period, pancreatitis presents additional challenges, as treatment options are often constrained by the patient’s ongoing physical recovery from the stresses of childbirth.

We present a case of a 22-year-old primigravida who developed an infected pancreatic pseudocyst four weeks after a cesarean section due to pregnancy complicated by HELLP syndrome. This case highlights the complex interaction between HELLP syndrome, shock, and the development of an infected pancreatic pseudocyst - a rare but serious complication - and also highlights the need for a balanced treatment approach that minimizes further stress on the patient while effectively managing this life-threatening condition.

## Case presentation

The patient, a 22-year-old primary gravida at 35 + 1 weeks [according to the last menstrual period (LMP)] with a previously uncomplicated pregnancy, presented to the Department of Gynecology and Obstetrics, with symptoms of headache and yellowish discoloration of sclera alongside elevated pressure (160/90 mmHg). Her liver function tests showed significantly elevated enzymes [bilirubin: 9.5 mg/dl, alanine transaminase (ALT): 294 U/L, aspartate transaminase (AST): 416 U/L, alkaline phosphatase (ALP): 453 U/L, gamma-glutamyl transferase (GGT): 119 U/L], indicating liver involvement consistent with HELLP syndrome.

The patient's hemoglobin and platelet levels were also low (8.4 g/dl and 160 10^9^/L respectively), further confirming the diagnosis of HELLP syndrome. Other laboratory findings, including viral serology and lipid profile, were normal, ruling out alternative conditions. An obstetric ultrasound was performed, which showed a pregnancy of 34 + 6 weeks with a decreased amniotic fluid index (AFI) of 5.5 CM. Given these complications, she underwent a cesarean section and delivered a healthy female baby. Interoperative blood loss was estimated to be 1,500 ml. Postoperatively, her condition worsened, and she developed bleeding from the surgical wound, leading to shock, disseminated intravascular coagulation (DIC), and anuria. She was stabilized in the ICU after multiple transfusions and hemodialysis and discharged on the 15th postoperative day.

**Figure 1:**
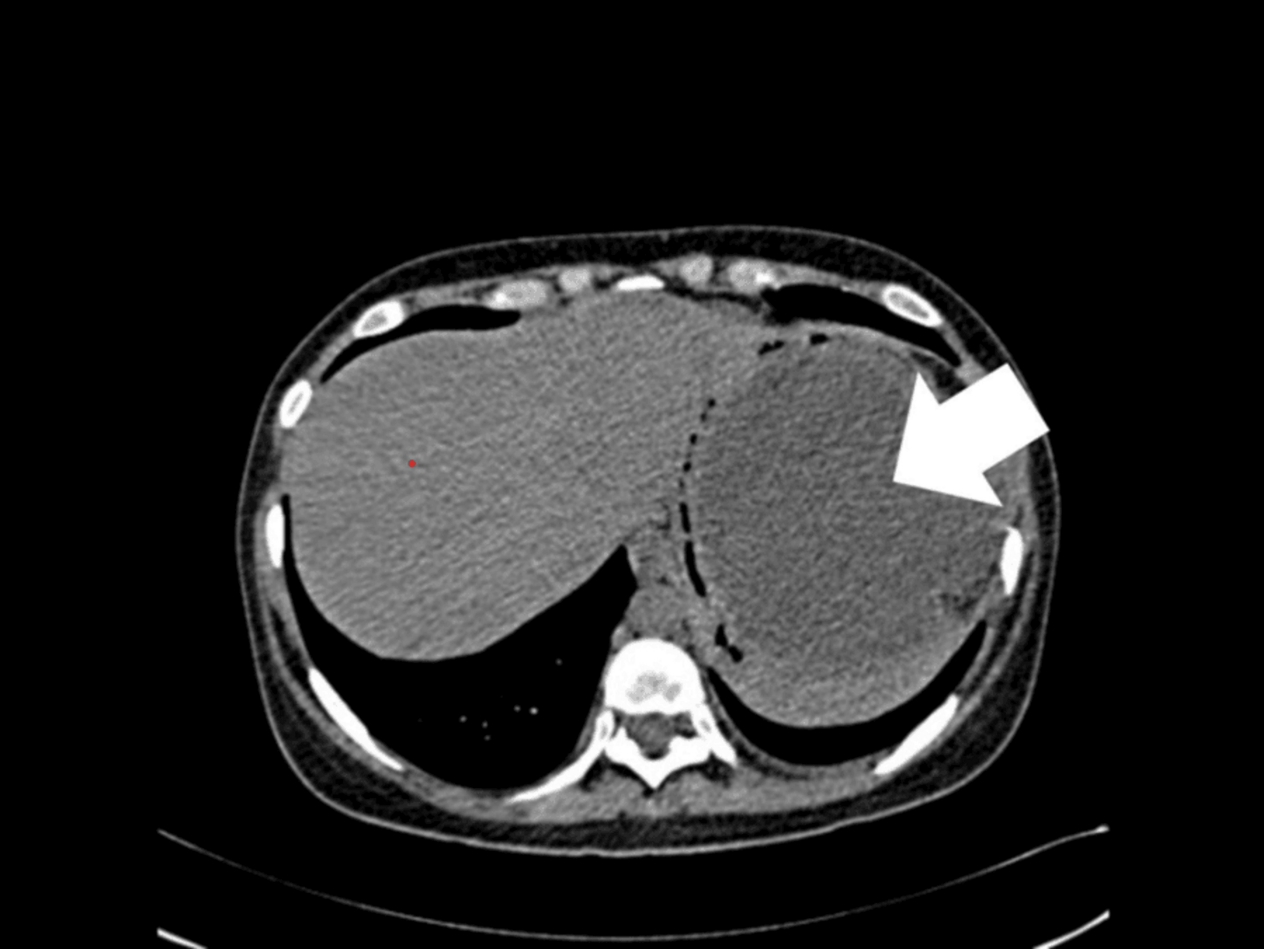
Triphase CT scan of the abdomen - axial view, non-contrast phase The image shows a large pancreatic pseudocyst in the left lateral retroperitoneal location measuring approximately 187 x 85 mm, encasing the distal pancreas with mildly dysmorphic pancreatic parenchyma suggestive of pancreatic pseudocyst CT: computed tomography

However, on the 27th postoperative day, the patient returned with a high fever (104 °F), tachycardia (120 beats per minute), pain, and swelling in the left lumbar region, as well as vomiting after eating. She was admitted to the surgical ward on the same day, samples were taken for laboratory investigations, an empirical antibiotic (ceftriaxone sodium 500 mg twice daily, intravenously) was started, and a CT scan was planned for the next day. CT scan was performed on the 28th day (Figure 1), which showed a large multiloculated collection in the left lateral retroperitoneal location measuring approximately 187 x 85 mm and extending from the left subdiaphragmatic location to the left pelvic region. This encased the distal pancreas with mildly dysmorphic pancreatic parenchyma, suggesting pancreatic pseudocyst. Serum amylase and lipase were done on the same day, which returned normal - amylase: 25 I/U (reference range: 23-85 I/U) and lipase 7 I/U (reference range: 0-160 I/U), indicating no signs of acute pancreatitis. 

The patient was continued on conservative management following the CT scan; she was administered broad-spectrum antibiotics and an ultrasound-guided drain was placed in the collection on the 29th post-operative day. Although 1500 ml of purulent fluid was drained, the patient continued to experience multiple fever spikes, prompting a shift to vancomycin on the 38th day (culture of fluid and blood showed no growth), which led to decreased inflammatory markers. The patient was also transfused three packed cell volumes (PCV) due to decreased hemoglobin levels of 6.2 g/dL. Serial ultrasounds were performed, which showed interval regression in the size of the collection. She was discharged on request on the 44th day with the drain in place and IV antibiotics (vancomycin 1 g) twice daily.

**Figure 2:**
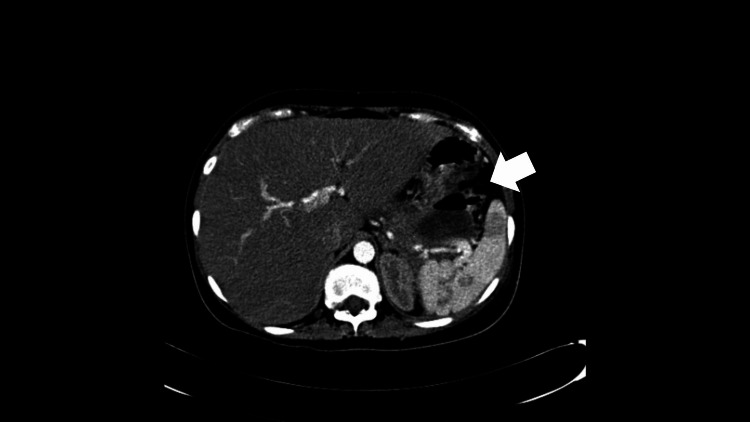
Triphase CT scan of the abdomen - axial view, arterial phase The image shows an organized heterogeneous left retroperitoneal collection with multiple loculations extending to the left pelvis with air specs tracking the skin over the left anterior superior iliac spine; a few air specks are also seen in the pancreatic tail CT: computed tomography

However, despite these efforts, the patient presented again on the 46th day with a fever (102 °F) and frank purulent discharge from skin near the left anterior superior iliac spine; a swab sample was taken from the discharge and microbiological analysis showed sensitivity to vancomycin. A repeat CT scan (Figure 2) showed a re-demonstration of a heterogeneous left retroperitoneal collection with multiple loculations extending to the left pelvis with air specs tracking the skin over the left anterior superior iliac spine, necessitating retroperitoneal percutaneous incision and drainage.

**Figure 3:**
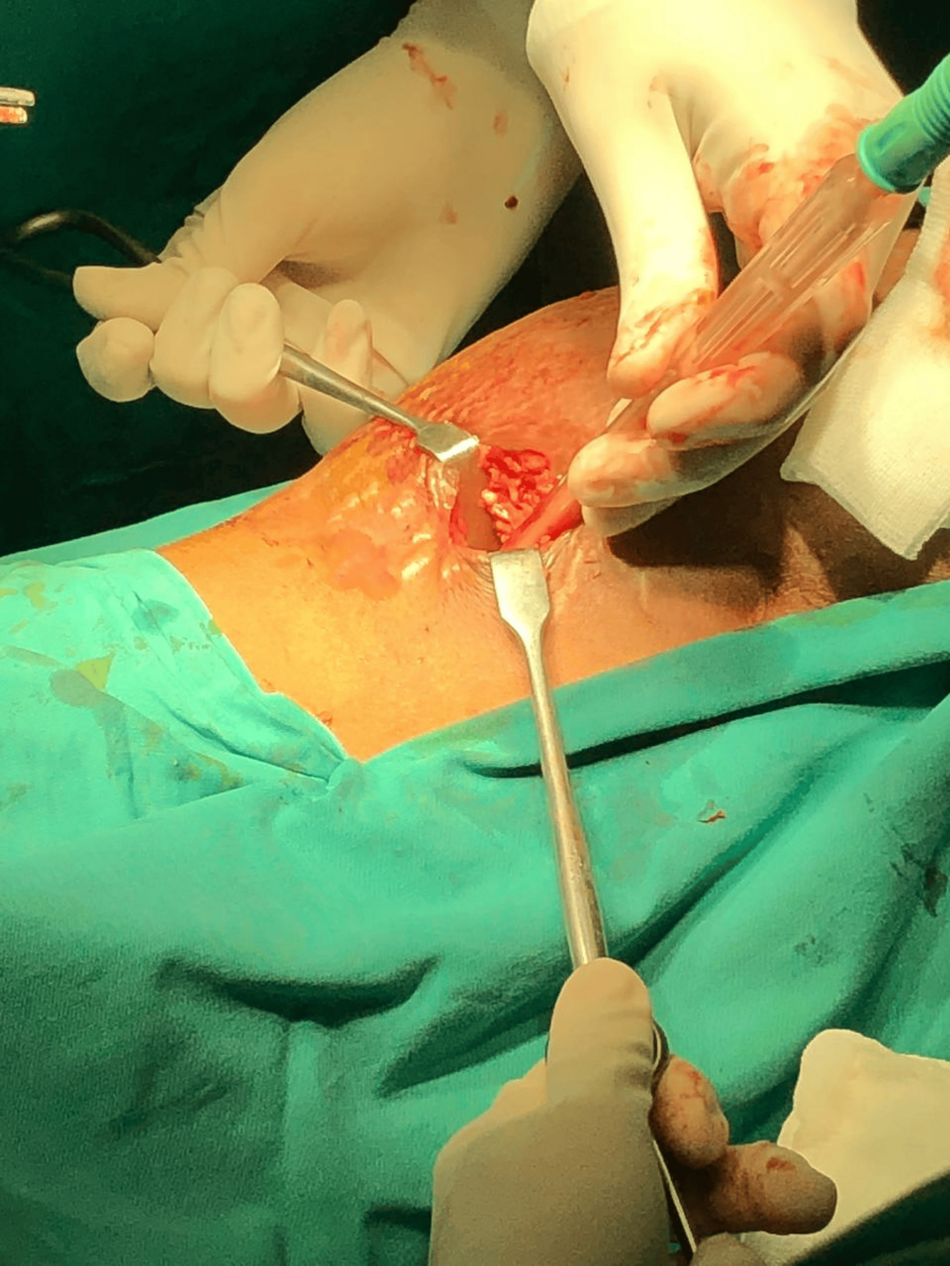
Minimally invasive retroperitoneal incision and drainage

The patient then underwent incision and drainage (Figure 3) via a retroperitoneal approach under general anesthesia on the same day. A skin incision was made near the left anterior superior iliac spine and 200 ml of pus was drained. Loculations were broken and necrotic pancreatic tissue was removed. The cavity was thoroughly washed with normal saline. Two corrugated rubber drains were placed.

Her wound and cavity were washed twice daily with normal saline. The fever resolved and inflammatory markers normalized. An ultrasound performed on day 51 showed edematous changes in the retroperitoneum but no fluid collection was noted. Her drains were removed and she was discharged on the same day with the advice of daily dressing. She remained on regular follow-ups, and complete healing with secondary intention was achieved in two weeks.

Table 1 below presents the results of the patient's laboratory tests at different time points after her cesarean section.

**Table 1:** Lab values at different time points after cesarean section The liver function tests and coagulation profile were deranged on day 1, indicating HELLP syndrome. Similarly, hemoglobin and platelet count decreased, and the coagulation profile deranged further along with an increase in values of renal function tests on the 2nd and 3rd days when the patient suffered from DIC and shock. The laboratory findings at the beginning of of the 2nd presentation, i.e., the 32nd postoperative day, showed the resolution of previous conditions (liver functions, renal function, and coagulation profile were normal). However, the total leukocyte count was elevated, indicating an infection, and the hemoglobin level was low, for which the patient was transfused 3 packed cell volumes. Infection was treated conservatively at first, with vancomycin, and an ultrasound-guided drain was placed to drain pus collection followed by minimally invasive incision and drainage. The findings on the day of discharge (51st postoperative day) showed improvement in hemoglobin levels as well as resolution of the infection

Variables	Reference ranges	Day 1	Day 2	Day 3	Day 32	Day 51
Hemoglobin	11.5–16.0 g/dL	8.4	6.3	7.2	6.7	9
Total leukocyte count (TLC)	4.0–11.0 10^9^/L	21.5	22.2	11.5	13.2	10.8
Platelets (PLT)	150–400 10^9^/L	160	120	256	544	664
Bilirubin	0.2 1.2 mg/dL	10.14	8.73	5.84	0.9	-
Alanine transaminase (ALT)	1–33 U/L	237	142	21	17	-
Aspartate aminotransferase (AST)	5–32 U/L	451	223	77	45	-
Alkaline phosphatase (ALP)	35–104 U/L	300	220	189	98	-
Total protein	6.4–8.3 g/dL	5	4.1	5.9	5.7	-
Albumin	3.5–5.2 g/dL	2.68	2.01	2.7	2.2	-
Globulin	2.5–3.5 g/dL	2.32	2.09	3.2	-	-
Albumin/globulin ratio	1.10–2.40 g/dL	1.16	0.96	0.84	-	-
Gamma-glutamyl transferase (GGT)	<40 U/L	73	55	126	41	-
Prothrombin time	11.0–14.0 seconds	23.6	36.6	-	15	-
Activated partial thromboplastin time (APTT)	25–35 seconds	39.5	>120	-	-	-
International normalized ratio (INR)	0.2–1.2 seconds	1.81	2.81	-	1.2	-
Glomerular filtration rate (GFR)	>60 mL/min	35.35	26.79	148	-	-
Urea	10–49 mg/dL	1.98	49	14	12	-
Creatinine	0.5–0.9 mg/dL	27	2.76	0.41	0.5	-

## Discussion

Acute pancreatitis is rare in pregnancy, affecting only 0.1-0.3% of the total pregnancy cases. Its presentation can range from mild pancreatitis to life-threatening complications like necrosis, abscess or pseudocyst formation, and multi-organ failure [2]. Advances in healthcare have helped decrease the mortality associated with pancreatitis in obstetrics patients from 50 to 5%. However, no proper treatment guidelines exist for treating pancreatitis in pregnant women [3].

Unlike non-pregnancy-induced pancreatitis, where the common causes are gallstones and alcohol consumption, biliary disease and hypertriglyceridemia account for 67% of the total cases in pregnancy [2-5,7]. However, our patient did not have any of the above-mentioned conditions, and hence the association between preeclampsia/HELLP syndrome, shock, and DIC with acute pancreatitis was considered. A study by Ramin and Ramsey showed no association between preeclampsia and pancreatitis [4], which contrasts with other studies, where a strong association was noticed between preeclampsia and acute pancreatitis [4,7-8]. Similarly, in another study, a critically ill patient with HELLP syndrome, DIC, and shock developed pancreatitis six weeks after hospital admission [9].

The exact pathophysiology behind preeclampsia and shock leading to pancreatitis is unknown, although both conditions are associated with microvascular abnormalities that can compromise blood flow and cause pancreatitis [9]. All these risk factors for developing pancreatitis were present in our patient, who had developed an infected pancreatic pseudocyst which was first seen on a CT scan four weeks after a complicated cesarean delivery. Our extensive literature research to explore the management options for infected pancreatic pseudocysts in the postpartum period [10-12] elicited only five cases of postpartum pancreatic pseudocyst formation, of which only one case was of infective etiology [10]. A review of all these case reports showed that the same management strategy was applied in pregnant women as that of the general population, with most cases treated conservatively [13].

According to the American Society of Gastroenterology best practice advice, a multi-disciplinary approach should be applied in the management of infective necrotizing pancreatitis. A conservative approach with broad-spectrum antibiotics and ultrasound-guided percutaneous drainage is the first-line treatment for septic pancreatitis [14-15]. Although endoscopic drainage is also a preferred technique, it is not suitable for critically ill patients and patients with infected collection at distant sites like paracolic gutters and pelvis [16]. These techniques, if not sufficient, are followed by surgical debridement after four weeks when the collection has walled off. This can be done using open necrosectomy, direct endoscopic necrosectomy, or minimally invasive techniques like laparoscopy or minimally invasive incision and drainage as in our case. This is also defined as a step-up approach by the American Society of Gastroenterology. It involves percutaneous drainage followed by minimally invasive retroperitoneal necrosectomy [17], which is considered the most suitable technique for infected pancreatic collections. This approach is less invasive and has led to a reduction in morbidity and mortality and a successful resolution of infected pancreatitis in many cases [17-18].

In our case, this highly conservative approach to the management of an infected necrotizing pancreatic pseudocyst after a complicated cesarean section was highly successful. Moreover, our patient was already recovering from a previous injury, and exposing her to any other highly invasive techniques that required anesthesia or surgery could have increased the risk of mortality and affected her well-being.

## Conclusions

This case highlights the successful management of postpartum infected pancreatic necrosis using a step-wise approach, starting with conservative treatment involving intravenous antibiotics and ultrasound-guided drainage followed by minimally invasive retroperitoneal incision and drainage. Given the unique challenges and risks associated with the postpartum period, we recommend that further guidelines be developed for obstetrics patients. Strategies using a step-wise approach should be considered in treating postpartum infected pseudocysts to improve outcomes in complex cases.
